# Symptom Prevalence of Anxiety and Depression in Older Cardiac Arrest Survivors: A Comparative Nationwide Register Study

**DOI:** 10.3390/jcm10184285

**Published:** 2021-09-21

**Authors:** Kristofer Årestedt, Johan Israelsson, Ingrid Djukanovic, Johan Herlitz, Jörg Carlsson, Suzanne Petersson, Anders Bremer

**Affiliations:** 1 Faculty of Health and Life Sciences, Linnaeus University, SE-39182 Kalmar, Sweden; johan.israelsson@regionkalmar.se (J.I.); ingrid.djukanovic@lnu.se (I.D.); jorg.carlsson@regionkalmar.se (J.C.); suzanne.petersson@regionkalmar.se (S.P.); 2 The Research Section, Region Kalmar County, Box 601, SE-39126 Kalmar, Sweden; 3 Section of Cardiology, Department of Internal Medicine, Region Kalmar County, Box 601, SE-39126 Kalmar, Sweden; 4 Faculty of Caring Science, Work Life and Social Welfare, University of Borås, SE-50190 Borås, Sweden; johan.herlitz@hb.se; 5 Department of Rehabilitation, Region Kalmar County, Box 601, SE-39126 Kalmar, Sweden; 6 Faculty of Health and Life Sciences, Linnaeus University, SE-35195 Växjö, Sweden; anders.bremer@lnu.se; 7 Department of Ambulance Service, Region Kalmar County, Box 601, SE-39126 Kalmar, Sweden

**Keywords:** aged, anxiety, depression, heart arrest, psychological distress

## Abstract

Knowledge about psychological distress in older cardiac arrest (CA) survivors is sparse, and the lack of comparisons with general populations make it difficult to draw any strong conclusions about prevalence and potential changes caused by CA. Our aim was to compare psychological distress between older CA survivors and a general population. This study included survivors 65–80 years old and an age- and sex-matched general population. Data on survivors was collected from the Swedish Register of Cardiopulmonary Resuscitation. The Hospital Anxiety and Depression Scale was used to measure psychological distress. Data were analyzed with non-parametric statistics. The final sample included 1027 CA survivors and 1018 persons from the general population. In both groups, the mean age was 72 years (SD = 4) and 28% were women. The prevalence of anxiety was 9.9% for survivors and 9.5% for the general population, while the corresponding prevalence for depression was 11.3% and 11.5% respectively. Using the cut-off scores, no significant differences between the groups were detected. However, CA survivors reported significantly lower symptom levels using the subscale scores (ΔMdn = 1, *p* < 0.001). In conclusion, the CA survivors did not report higher symptom levels of anxiety and depression than the general population. However, since psychological distress is related to poor quality-of-life and recovery, screening for psychological distress remains important.

## 1. Introduction

Cardiac arrest (CA) is a common health problem worldwide, especially in older people. In Europe, approximately 350,000 suffer out-of-hospital cardiac arrest (OHCA) annually [1,2], while the incidence of in-hospital cardiac arrest (IHCA) is estimated as 1–5 per 1000 hospital admissions [3,4]. The prognosis is poor, but the survival rates have improved during recent decades [5]. The increased number of survivors has drawn further attention to shortcomings in care and the need for evidence-based practice. Identification and treatment of psychological distress is of great importance, since such problems have an impact on health state, quality of life, and mortality [6,7]. According to the American Psychological Association, psychological distress is a state of emotional suffering characterized by symptoms of anxiety and depression, ranging from normal fluctuations to psychopathology. Psychological distress is often assessed by putative self-reported measures [8].

Psychological distress is commonly reported in CA survivors [9,10]. A review study by Wilder Schaaf et al. reported a prevalence of 13–61% for symptoms of anxiety and 14–45% for symptoms of depression in OHCA survivors [9]. The prevalence of psychological distress in IHCA survivors has not been studied to the same extent, however a Swedish register study by Israelsson et al. reported a prevalence of 15% and 13% for symptoms of anxiety and depression, respectively [11]. The variations in prevalence across studies can probably be explained by heterogeneity in the inclusion criteria, definitions, and measures. However, these studies have not determined if psychological distress is a consequence of suffering a cardiac arrest. In addition, there is a lack of comparison studies including general populations.

Anxiety and depression are common in older people in general [12,13,14,15]. The average age among those suffering cardiac arrest varies between countries and studies, but a mean age of 65 years or higher is commonly reported [2,16]. Despite this, existing studies about psychological distress are commonly based on age-heterogeneous samples [9]. However, CA probably affects people’s lives differently during the life cycle, leading to a need for studies focusing on psychological distress in older CA survivors.

In summary, the knowledge about psychological distress in older CA survivors is sparse and the lack of comparisons with general populations make it difficult to draw any strong conclusions about prevalence and potential changes caused by the CA. Therefore, the aim of this study was to compare psychological distress between older CA survivors and an age- and sex-matched general population.

## 2. Materials and Methods

### 2.1. Design

This comparative study was based on data from the Swedish Register of Cardiopulmonary Resuscitation (SRCR) collected between 2014 and 2017 and an age- and sex-matched sample from a cross-sectional survey including a random sample of individuals from the total Swedish population in the age group 65–80 years [13]. Ethical approval was obtained from the Regional Ethical Review Board in Stockholm, Sweden (No. 2010/823-314/4, No. 2017/7988-32).

### 2.2. Sample and Procedure

The inclusion of CA survivors was performed using register data from the SRCR. This registry covers a vast majority of CAs in Sweden. Data for the SRCR is collected at three times: (1) at the time of the arrest, (2) 30-days post arrest, and (3) 3–6 months post arrest. The first and second registrations include information about, for example, prearrest conditions, aetiology, treatments, survival, and neurological function (Cerebral Performance Category scale = CPC). Inclusion criteria for the third registration are to be alive 3–6 months after the CA and ≥18 years of age. Unwillingness to participate, severe cognitive dysfunction and language difficulties are criteria for exclusion. An invitation to a telephone follow-up interview is offered and a questionnaire is sent to all CA survivors who conform to the selection criteria. The questionnaire includes the Hospital Anxiety and Depression Scale (HADS) and other patient reported outcome measures. The CA survivors receive instructions to complete the questionnaire during the interview, which is performed by resuscitation coordinators or cardiac rehabilitation nurses. The present study included CA survivors 65 to 80 years old, registered between 2014 and 2017. During this period, the response rate for all CA survivors was 56%.

The general population sample was taken from a national survey investigating psychological distress in the age group 65–80 years. The survey was conducted as a postal questionnaire including demographic data and symptoms of anxiety and depression measured with HADS. In total, 6659 randomly selected participants were included during 2010. More details of the study are presented in Djukanovic et al. [13]. To make the two samples comparable, the samples were stratified according to sex and age (five-year intervals). Participants from the general population sample were then randomly selected from each stratum to reach equal distributions of age and sex between the two samples.

### 2.3. Measures

The HADS is a self-rating scale developed to assess psychological distress in non-psychiatric patients and consists of two subscales, anxiety (HADS-A) and depression (HADS-D) [17]. The HADS consists of 14 items, seven for measuring symptoms of anxiety and seven for symptoms of depression. Each item is scored on a response-scale with four alternatives, ranging from 0 to 3. The item responses within each subscale are summarized to a total score with a possible range between 0 and 21. A higher score indicates a more severe problem with anxiety and/or depression. Snaith has recommended the following cut-off scores: 0–7 normal range, 8–10 suggested presence of mood disorder (minor problems), and 11–21 probable presence of mood disorder (moderate to major problems) [18]. The HADS is not validated in CA survivors but is recommended by the American Heart Association [19] and the European Resuscitation Council [3] to screen for symptoms of anxiety and depression among these patients. It has also demonstrated satisfactory measurement properties in related groups, e.g., patients with heart disease [20] and general populations of elderly people [21]. The subscale scores with respective cut-off scores were used in the present study. In CA survivors, internal consistency as measured by Cronbach’s alpha was 0.89 for HADS anxiety and 0.85 for HADS depression in the present study. The corresponding alpha values for the general population used in this study were 0.85 and 0.81, respectively.

### 2.4. Statistical Analyses

The CA survivors had no missing data in HADS. In contrast, 57 observations from the general population had missing data, ranging from 1 to 14 missing values. Missing data were imputed using the subscale half mean procedure recommended by Bell et al. [22], i.e., data were replaced using person mean score in observations with ≤3 missing values for each subscale, respectively. In total, 80 missing values were imputed for 48 observations. Consequently, nine observations were excluded. Thus, the statistical analyses regarding HADS were conducted on 1027 CA survivors and 1018 persons from the general population.

Descriptive statistics were used to present sample characteristics and symptoms of anxiety and depression. The independent sample t-test and Pearson chi-square test were conducted to compare differences in age and gender between the CA survivors and the general population.

In order to compare differences in psychological distress, the analyses were conducted in two steps. In the first step, both IHCA and OHCA survivors were treated as one group and compared with the general population. Based on the HADS subscale scores, the Mann–Whitney U test was used to compare differences in anxiety and depression. With HADS subscales grouped into three categories (normal, suggested and probable presence of anxiety and depression), the Pearson chi-square test was used. In the second step, CA survivors were regrouped by IHCA and OHCA and compared with the general population. Based on the HADS subscale scores, the Kruskal–Wallis test was conducted to compare differences in psychological distress between the three groups. The Mann–Whitney U test was used as a post-hoc test and the Bonferroni correction was applied to reduce the likelihood of wrongly rejecting the null hypothesis (i.e., type I error). The Pearson chi-square test was conducted to compare differences in anxiety and depression between the three groups, using the same categorization of the HADS subscale described above.

The level of statistical significance was set at *p* < 0.05, except for the Bonferroni correction in the post-hoc analyses where the significance level was set at *p* < 0.017. All statistical analyses were conducted in Stata 16.1 (StataCorp LLC, College Station, TX, USA).

## 3. Results

### 3.1. Sample Characteristics

The characteristics of the participants are presented in Table 1. The mean age was 71.9 (SD = 4.4) in the CA survivors and 71.8 (SD = 4.4) in the general population. The gender distribution was equal in both groups, with 736 (71.7%) men and 291 (28.3%) women in the CA survivors and 730 (71.7%) men and 288 (28.3%) women in the general population. No significant difference was shown in age (*p* = 0.66) or sex (*p* = 0.98) between the groups. A majority of the survivors had suffered IHCA (*n* = 694, 67.6%), had a cardiac aetiology (*n* = 746, 72.6%), had a shockable initial rhythm (ventricular fibrillation or ventricular tachycardia, *n* = 608, 59.2%), and were defibrillated (*n* = 636, 61.9%). The CA was witnessed in 956 cases (93.1%). The most common place for OHCA was the survivor’s own home (*n* = 140, 42.0%), while the most common places for IHCA were heart intensive care units (*n* = 165, 23.8%) and general wards (*n* = 165, 23.8%). According to the CPC scale, most survivors had good neurological function (CPC 1) at inclusion (*n* = 886, 86.3%).

### 3.2. Comparisons of Psychological Distress between CA Survivors and the General Population

No significant differences were detected between CA survivors and the general population using the cut-off scores for anxiety (*p* = 0.701) and depression (*p* = 0.703). Of the CA survivors, 9.9% (*n* = 102) reported suggested or probable presence of anxiety and 11.5% (*n* = 118) reported suggested or probable presence of depression. The corresponding prevalence in the general population was 9.5% (*n* = 97) for anxiety and 11.3% (*n* = 115) for depression (Table 2).

Based on the HADS subscale scores, both CA survivors and the general population generally reported low levels of both anxiety and depression. The general population reported significantly higher levels of symptoms in both anxiety (ΔMdn = 1, *p* < 0.001) and depression (ΔMdn = 1, *p* < 0.001) compared with the CA survivors (Table 2).

### 3.3. Comparisons of Psychological Distress between IHCA Survivors, OHCA Survivors, and the General Population

No significant differences were detected between IHCA survivors, OHCA survivors, and the general population using the cut-off scores for anxiety (*p* = 0.190) and depression (*p* = 0.489). Of the IHCA survivors, 11.4% (*n* = 79) reported suggested or probable presence of anxiety and 12.5% (*n* = 87) reported suggested or probable presence of depression. The corresponding prevalence in OHCA survivors was 6.9% (*n* = 23) for anxiety and 9.3% (*n* = 31) for depression (Table 3).

The comparison of the HADS subscale scores between IHCA survivors, OHCA survivors and the general population showed significant differences overall in anxiety (ΔMdn = 1, *p* < 0.001) and depression (ΔMdn = 2, *p* < 0.001) between the three groups. The post hoc analysis showed that the general population scored significantly higher symptom levels in both anxiety and depression compared with OHCA and IHCA survivors. In addition, IHCA survivors scored significantly higher symptom levels in anxiety and depression compared with OHCA survivors (Table 3).

## 4. Discussion

To the best of our knowledge, this is the first large study that has compared psychological distress between older CA survivors and a general population matched for age and sex. Overall, the present study showed that the CA survivors reported lower or equal levels of psychological distress compared with the general population.

Using the HADS subscale scores, CA survivors reported significantly lower symptom levels in both anxiety and depression. However, the effect size was small and no differences were detected using the cut-off scores. This indicates that the differences between the two groups are related to the lower end of the subscales, i.e., the part of the scale that is defined as the normal range by Snaith [18]. Our findings may implicate problems with using ≥8 as a cut-off score in CA survivors. A review by Bjelland et al. [23] shows that different cut-off scores have been suggested for HADS across studies and in different samples. They concluded that values ≥8 are commonly recommended, but they also showed that lower cut-off scores can be of importance. Individual studies in the review suggested ≥3 for anxiety in primary care [24] and ≥4 for depression in post stroke care [25]. We have also previously reported that one single question about anxiety/depression in EQ-5D-5L identified a higher prevalence of psychological distress compared with ≥8 as a cut-off score for HADS [11]. Lack of evidence of a clinically relevant cut-off score for CA survivors may result in poor sensitivity and specificity, and further studies are therefore needed. Until then, HADS subscale scores should be reported in addition to the cut-off scores.

The CA survivors did not report more problems with anxiety and depression compared with the general population. These results are partly supported by a previous study by Israelsson et al. who reported equal levels of psychological distress, measured by a single item from EQ-5D, in ICD-implanted CA survivors and an age matched general population. They also found that the CA survivors reported better general health [26]. Similar findings have also been reported in other patient groups who have survived a life-threatening illness, for example cancer [27]. One possible explanation could be gratitude towards being alive and a re-evaluation of living-preferences [28,29]. In addition, after the first European guidelines for post CA follow-up care were published in 2015 [30], improvements in care might also have contributed by improving the screening for psychological distress and referral to specialized psychological care when needed. In contrast, people from the general population need to seek help themselves, and it is well known that stigma is an important barrier for help-seeking [31]. For example, a population-based study of older persons in Sweden showed that only 2.9% of those who scored suggested or probable presence of depression using HADS visited a psychologist [13]. However, these mechanisms are not well explored in CA survivors, and the interpretation of the findings are complicated by the fact that few studies have data on psychological distress prior to CA.

It is important to remember that the present study included only older CA survivors and the mean age was therefore high compared with previous studies. Thus, the findings from the present study might not be comparable with studies that also included younger CA survivors. For example, in the present study both the CA survivors and the general population reported a higher prevalence of depression than anxiety. This is in contrast to studies reporting similar [32] or higher prevalence of anxiety compared with depression in CA survivors [10]. Hence, the state of knowledge is unclear and there is a need for further studies on the potential importance of age in relation to anxiety and depression in this group. However, as previous studies have shown that psychological distress is associated with recovery and quality of life [10,33], the importance of screening remains.

## 5. Methodological Limitations

The present study has some important limitations that need to be considered. We used data from one of the largest Swedish surveys in order to have a valid comparison group representing a general population of older people in Sweden. Since the survey was restricted to people 65 to 80 years old, CA survivors in the same age span were included. The age of 65 years is commonly used in studies of older people in Sweden, since this is the official age for retirement. However, the upper restriction of 80 years is somewhat problematic, as the findings will not reflect the situation among older people.

The response rate for all CA survivors in the SRCR during 2014–2017 was 56%, and along with the unwillingness of some survivors to participate in the study, selection bias can thus not be excluded. Data collection for the SRCR requires that the survivors themselves can read and complete the self-reported instruments, which excludes persons with severe cognitive decline. Since cognitive impairments are common in CA survivors [34], and are also related to mental health [10], it is likely that psychological distress in this group is underestimated. However, this possible selection bias is probably present in the general population as well, since data collection was conducted in a similar way with postal questionnaires. Related to this, the lack of sufficient background data on both groups, such as co-morbidity and socioeconomic situation, implies uncertainty regarding group comparability. Thus, the findings should be generalized with some caution.

In the present study, symptoms of anxiety and depression were measured as two separate aspects of psychological distress. Generally, anxiety and depression have a high degree of symptom overlap, and co-morbidity with other affective or mood disorders is common. About 50% of those with an anxiety or depression diagnosis have shown to have one or more additional affective/mood diagnoses [35,36]. It has also been suggested that different symptoms of psychiatric distress could be various expressions for one disorder [37]. Despite this, the HADS is widely used with its two original scales and has thus shown good psychometric properties [21,23]. Different cut-off scores have been suggested in previous research studies. In the present study, we used one of the most common cut-off scores for the HADS, as suggested by its creator [18,23]. However, the instrument and the cut-off scores have not been validated in CA survivors. This is a limitation for the present study, as it is for other CA studies based on HADS.

## 6. Conclusions

Previous research studies have often described psychological distress as a common problem in CA survivors, and screening is recommended in international clinical guidelines. However, when using a general population comparison group, the survivors in the present study did not report higher symptom levels of anxiety or depression. Since psychological distress is related to poor quality of life and recovery, it is important from a clinical perspective to identify CA survivors with these problems. Thus, screening for psychological distress remains important.

## Figures and Tables

**Table 1:** Sample characteristics.

	CA Survivors, *n* = 1027	General Population, *n* = 1018	*p*-Value
Age, mean (SD)	71.9 (4.4)	71.8 (4.4)	0.658
Sex, *n* (%)			0.982
Woman	291 (28.3)	288 (28.3)	
Man	736 (71.7)	730 (71.7)	
Etiology, *n* (%)			
Cardiac	746 (72.6)		
Other	268 (26.1)		
Unknown	13 (1.3)		
Initial rhythm, *n* (%)			
VF/VT	608 (59.2)		
PEA	95 (9.3)		
Asystole	156 (15.2)		
Unknown	168 (16.4)		
Place of CA, *n* (%)			
IHCA			
Heart intensive care unit	165 (16.1)		
Angiography laboratory	143 (13.9)		
Intensive care unit	60 (5.8)		
Emergency care unit	82 (8.0)		
Intermediate care	7 (0.7)		
Surgical ward	21 (2.0)		
General ward	165 (16.1)		
Reception ward	33 (3.2)		
Other within hospital	18 (1.8)		
OHCA			
Home	140 (13.6)		
Public place	122 (11.9)		
Other out of hospital	71 (6.9)		
Witnessed, *n* (%)			
Yes	956 (93.1)		
No	62 (6.0)		
Unknown	9 (0.9)		
Defibrillation, *n* (%)			
Yes	636 (61.9)		
No	384 (37.4)		
Unknown	7 (0.7)		
CPC at inclusion, *n* (%)			
CPC 1 (good performance)	886 (86.3)		
CPC 2 (moderate disability)	110 (10.7)		
CPC 3 (severe disability)	20 (2.0)		
Unknown	11 (1.1)		

CA = cardiac arrest; CPC = cerebral performance category; IHCA = in-hospital cardiac arrest; OHCA = out-of-hospital cardiac arrest; PEA = pulseless electrical activity; VT/VF = ventricular tachycardia/ventricular fibrillation.

**Table 2:** Comparisons of psychological distress between cardiac arrest survivors and a general population matched for age and sex.

	CA Survivors, *n* = 1027	General Population, *n* = 1018	*p*-Value
Anxiety score, Mdn (q1–q3)	1 (0–4)	2 (1–5)	<0.001 ^a^
Anxiety categorized, *n* (%)			0.701 ^b^
Normal range (scores 0–7)	925 (90.1)	921 (90.5)	
Suggested presence (scores 8–10)	64 (6.2)	66 (6.5)	
Probable presence (scores 11–21)	38 (3.7)	31 (3.0)	
Depression score, Mdn (q1–q3)	2 (0–4)	3 (1–5)	<0.001 ^a^
Depression categorized, *n* (%)			0.703 ^b^
Normal range (scores 0–7)	909 (88.5)	903 (88.7)	
Suggested presence (scores 8–10)	75 (7.3)	79 (7.8)	
Probable presence (scores 11–21)	43 (4.2)	36 (3.5)	

CA = cardiac arrest; ^a^ Mann-Whitney U test; ^b^ Pearson chi-square test.

**Table 3:** Comparisons of psychological distress between in-hospital cardiac arrest survivors, out-of-hospital cardiac arrest survivors and a general population matched for age and sex.

	Survivors, IHCA, *n* = 694	Survivors, OHCA, *n* = 333	General Population, *n* = 1018	*p*-Value	Post-Hoc Test ^†^
Anxiety score, Mdn (q1–q3)	2 (0–5)	1 (0–3)	2 (1–5)	<0.001 ^a^	ABC
Anxiety categorized, *n* (%)				0.190 ^b^	
Normal range (scores 0–7)	615 (88.6)	310 (93.1)	921 (90.5)		
Suggested presence (scores 8–10)	51 (7.4)	13 (3.9)	66 (6.5)		
Probable presence (scores 11–21)	28 (4.0)	10 (3.0)	31 (3.0)		
Depression score, Mdn (q1–q3)	2 (1–5)	1 (0–3)	3 (1–5)	<0.001 ^a^	ABC
Depression categorized, *n* (%)				0.489 ^b^	
Normal range (scores 0–7)	607 (87.5)	302 (90.7)	903 (88.7)		
Suggested presence (scores 8–10)	57 (8.2)	18 (5.4)	79 (7.8)		
Probable presence (scores 11–21)	30 (4.3)	13 (3.9)	36 (3.5)		

IHCA = in-hospital cardiac arrest; OHCA = out-of-hospital cardiac arrest; ^a^ Mann-Whitney U test; ^b^ Pearson chi-square test. ^†^ Post-hoc test based on Mann-Whitney U test with Bonferroni corrected *p*-values (*p* < 0.017): A = IHCA vs. OHCA, B = IHCA vs. general population, C = OHCA vs. general population.

## Data Availability

Data are available from the Swedish Register of Cardiopulmonary Resuscitation (SRCR) and the corresponding author upon reasonable request.
